# Raslan Circuit: von Economo neurons as biological dipole antenna elements in a proposed FI–ACC communication pathway for multimodal integration and conscious access

**DOI:** 10.3389/fnsys.2026.1779601

**Published:** 2026-08-21

**Authors:** Mohamed Khaled Younes Raslan

**Affiliations:** Urology Specialist, Private Clinic, Jeddah, Saudi Arabia

**Keywords:** anterior cingulate cortex, consciousness, electromagnetic field, ephaptic coupling, fronto-insular cortex, terahertz, von Economo neurons, working memory

## Abstract

Consciousness remains incompletely explained at the anatomical, biophysical, and functional levels. Although existing theories characterize key functional correlates of conscious experience—including global broadcasting, information integration, attentional selection, working-memory-based awareness, and electromagnetic field dynamics—none has yet identified a specific cellular mechanism embedded within a defined long-range cortical pathway that could support the transmission of integrated sensory, interoceptive, emotional, and cognitive information toward anterior access and control systems. Here, the “Raslan Circuit” is defined as a candidate solution centered on the fronto-insular cortex (FI), the anterior cingulate cortex (ACC), and their von Economo neurons (VENs). Anatomically, VENs form a selectively localized population in FI and ACC, two regions already implicated in salience processing, interoception, emotional evaluation, and conscious awareness. Biophysically, within the Raslan Circuit hypothesis, VENs are proposed to act not merely as histological markers of higher cognition but as elongated, polarity-organizing neuronal elements with biological dipole antenna properties, supporting field-sensitive coupling and potentially resonance-based communication between FI and ACC. Functionally, FI is proposed to act as a deep multimodal collecting and organizing station, while ACC serves as a receiving and redistributive hub that links the transmitted signal to working memory, awareness, emotional valuation, and action selection. Together, this FI–ACC–VEN axis is advanced as a previously unrecognized communication infrastructure that could complement—rather than replace—Global Workspace Theory, Integrated Information Theory, embedded-processes and awareness-buffer models of working memory, salience-network accounts, and electromagnetic field theories of consciousness. The dimensional properties of VENs suggest a theoretically motivated, though speculative, prediction of resonance in the terahertz range, providing a falsifiable spectral hypothesis. More generally, the Raslan Circuit is grounded in four interlocking components: an anatomical foundation in VEN-rich FI and ACC, a biophysical rationale based on field-sensitive coupling, an explicit integration with existing functional theories of consciousness, and a set of testable predictions spanning connectivity, cellular and field measurements, clinical syndromes, and computational modeling.

## 1 Introduction

The insular cortex occupies a paradoxical position in human neuroanatomy. Located deep within the lateral sulcus and concealed beneath the frontal, parietal, and temporal opercula, it is not visible on the external surface of the brain without surgical retraction or removal of the overlying cortex. This anatomical concealment may have contributed, at least in part, to the relative underrepresentation of the insula in classical models of consciousness and large-scale brain communication. Namkung et al. (2017) described the insula as an underestimated brain area in clinical neuroscience, psychiatry, and neurology, a characterization that is particularly relevant to the present hypothesis. Yet the insula is surrounded by cortical and subcortical territories that process sensory, interoceptive, autonomic, emotional, and cognitive information across multiple modalities. It receives projections from posterior sensory and association cortices, from limbic and paralimbic structures, from thalamic relay nuclei, and from prefrontal control systems. Far from being a peripheral or specialized cortical region, the insula is therefore positioned at the anatomical intersection of systems involved in external perception, internal bodily state, affective valuation, and cognitive control.

Despite major theoretical advances, the neuroscience of consciousness still lacks a complete anatomical and biophysical account of how distributed information becomes consciously accessible. Existing theories have made substantial contributions. Global Workspace Theory describes how information becomes globally available once a threshold of activation is reached (Dehaene et al., 2011); Information Integration Theory formalizes the relationship between integration and conscious experience (Tononi, 2004); salience-network models identify the fronto-insular and anterior cingulate cortices as key nodes for detecting behaviorally relevant events and coordinating access to attentional and control resources (Seeley et al., 2007; Menon and Uddin, 2010); and electromagnetic field theories propose that conscious experience may be related to specific patterns of brain-generated electromagnetic activity. Working memory models, including the recent awareness-buffer reformulation, further emphasize conscious access as a limited-capacity multimodal interface. Nevertheless, none of these frameworks has identified a specific cellular mechanism embedded within a defined long-range cortical pathway that might physically carry integrated information toward anterior access and control systems.

Within the insular cortex and its immediate anterior cingulate counterpart, one neuronal class stands out for its morphological distinctiveness and selective distribution: the von Economo neuron (VEN). First systematically described by Constantin von Economo and Georg Koskinas in the 1920s, VENs are large, elongated, stick-like or corkscrew-like neurons located predominantly in layer Vb of the fronto-insular cortex (FI) and the anterior cingulate cortex (ACC). Their unusual morphology, laminar specificity, and selective presence in precisely the two cortical regions involved in salience, interoception, emotional awareness, and cognitive control suggest that they may represent a cellular specialization of the FI–ACC axis rather than a histological curiosity. The present paper proposes that VENs are not merely markers of higher-order cortical organization, but biological dipole antenna elements—that is, neuronal structures whose elongated bipolar morphology may equip them for directed electromagnetic communication between FI and ACC.

This proposed framework is designated Raslan Circuit. The term is used here as a specific label for a hypothesized FI–ACC–VEN communication pathway that has not, to current knowledge, been previously described in this form in the scientific literature. The name is not intended to imply that the circuit operates in isolation or that it constitutes a complete theory of consciousness. Rather, Raslan Circuit is proposed as a specific anatomical and biophysical mechanism that may complement existing frameworks by identifying a candidate cellular substrate (VENs), a candidate anatomical route (the FI–ACC axis), and a candidate biophysical mechanism (antenna-like field organization and resonance-based coupling) for the transmission of integrated multimodal information toward anterior conscious-access systems.

The present paper develops this hypothesis through four main components. First, it outlines the anatomical foundations of Raslan Circuit, including the fronto-insular cortex, the anterior cingulate cortex, and the von Economo neurons. Second, it examines the electromagnetic and biophysical rationale, including the distinction between electric dipoles and antenna dipoles, the relevance of ephaptic coupling, and the spectral predictions generated by the model. Third, it situates the proposed circuit within broader functional theories of consciousness, working memory, and salience. Fourth, it describes the empirical predictions, limitations, and possible clinical implications that follow from the hypothesis.

## 2 Anatomical foundation of Raslan Circuit

### 2.1 The insula as a hidden anatomical gateway

The insular cortex is a deep cortical structure located within the lateral sulcus. Unlike the frontal, parietal, temporal, and occipital lobes, the insula is not visible on the external surface of the brain unless the frontal, parietal, and temporal opercula are retracted or removed. These opercula form a cortical cover over the insula, giving it the appearance of a hidden cortical island. This deep and concealed position is anatomically important for the Raslan Circuit hypothesis because it places the insula in a location that is not superficially obvious, yet is surrounded by cortical and subcortical systems involved in multiple domains of information processing.

Grossly, the insula is divided by the central sulcus of the insula into anterior and posterior parts. The anterior insula contains the short insular gyri, while the posterior insula contains the long insular gyri. The inferior anterior part of the insula, known as the limen insulae, represents a transition zone between the insular surface and deeper basal forebrain structures. This region is especially relevant to the present hypothesis because it lies near the anterior perforated substance and deep basal anatomical corridors. Therefore, the insula is not only hidden within the lateral sulcus; it also occupies a deep transitional position between cortical, subcortical, limbic, and vascular anatomical domains.

The cytoarchitecture of the insula further supports its role as a transitional and integrative cortical region. The posterior insula is generally more granular and is closely related to sensory and interoceptive representation, whereas the anterior insula is more dysgranular and agranular and is more strongly associated with salience, awareness, emotional integration, autonomic regulation, and cognitive control. This anterior–posterior gradient suggests that information may move from more sensory and bodily representations posteriorly toward more integrated subjective and cognitive representations anteriorly. In the present framework, the anterior fronto-insular region is not treated as a passive cortical area. It is interpreted as a deep collecting and organizing station positioned near the convergence of external sensory information, internal bodily signals, affective valuation, salience detection, and cognitive control.

### 2.2 Von Economo neurons: a unique neuronal class with distinctive morphology and location

The first cellular anchor of the Raslan Circuit hypothesis is the von Economo neuron. Before discussing any proposed electromagnetic or circuit-level role, it is essential to define these cells precisely, because the term “von Economo neuron” has sometimes been used inconsistently in the literature. In the present paper, the term is used in a conservative anatomical sense, referring primarily to the specialized neurons originally described by von Economo in the human anterior cingulate cortex and fronto-insular cortex.

While studying the cytoarchitecture of the human cerebral cortex, von Economo noticed a peculiar population of large elongated neurons in the anterior cingulate cortex and the fronto-insular cortex. Later, the systematic cortical analysis of von Economo and Koskinas using Nissl staining showed that these cells had a highly distinctive morphology (von Economo and Koskinas, 1925); an extremely elongated stick-like or corkscrew-like soma, located mainly in layer Vb of the ACC and FI, often arranged in small clusters. Their unusual shape was so striking that von Economo initially considered them a possible pathological alteration, before they were later recognized as a specialized neuronal subtype.

This point is important because these cells are not simply ordinary spindle-shaped neurons. Common fusiform or spindle-shaped neurons can be found in many cortical areas, particularly in deeper cortical layers. However, von Economo explicitly distinguished his specialized stick-like or corkscrew-like cells from the more common spindle-shaped neurons found throughout the cerebral cortex. Therefore, Raslan Circuit is not based on any elongated neuron found anywhere in the cortex, but specifically on the specialized FI–ACC neuronal population originally described by von Economo and Koskinas.

The terminology surrounding these cells has contributed to later confusion. Some early modern studies referred to the specialized stick-corkscrew neurons as spindle cells, whereas later studies increasingly used the term von Economo neurons. Using spindle cells and von Economo neurons interchangeably may obscure an important anatomical distinction: not every spindle-shaped or fusiform neuron is necessarily a true von Economo neuron.

For this reason, the present paper adopts a strict definition. It focuses on von Economo neurons as specialized stick-like or corkscrew-like neurons located mainly in layer Vb of the anterior cingulate cortex and fronto-insular cortex. Although recent studies have reported von Economo-like or spindle-shaped cells in additional cortical regions, species, and layers, these reports do not necessarily prove that such cells are identical to the specialized neurons originally described by von Economo. This conservative definition matters because the proposed circuit depends on the specific anatomical relationship between FI, ACC, and their specialized VEN population.

The regional location of these cells is not incidental. The anterior cingulate cortex and fronto-insular cortex are both involved in salience processing, interoception, emotional evaluation, autonomic regulation, cognitive control, attention, and conscious awareness. The presence of a distinctive neuronal class in precisely these two regions suggests that VENs may represent a cellular specialization of the FI–ACC axis.

### 2.3 Von Economo neurons as biological dipole antennas

After defining von Economo neurons as a specialized neuronal class, the next step is to explain their proposed biophysical role within Raslan Circuit. In this hypothesis, von Economo neurons are proposed to function as biological dipole antennas. This interpretation is based on their distinctive morphology, their bipolar-like organization, their deep layer Vb position, and their selective distribution within the fronto-insular cortex and anterior cingulate cortex.

The morphology of VENs is central to this proposal. These cells are described as large, elongated, stick-like or corkscrew-like neurons, clearly distinct from ordinary pyramidal neurons and from common spindle-shaped or fusiform neurons. Their long, narrow, vertically oriented soma gives them a polarized architecture. This structure allows the cell to be interpreted as a biological dipole: a cellular element with two opposite anatomical poles capable of organizing electrical activity along its longitudinal axis.

In a classical dipole antenna, two aligned conductive arms allow oscillating electrical activity to be transmitted or received as electromagnetic waves. In the Raslan Circuit hypothesis, the elongated bipolar-like structure of VENs provides the biological equivalent of this geometry. VENs are therefore proposed not only as projection neurons for synaptic communication, but also as antenna-like neuronal elements capable of transmitting and receiving electromagnetic information within the brain.

This antenna interpretation is strengthened by the anatomical distribution of VENs. These neurons are concentrated in two key cortical regions: the fronto-insular cortex and the anterior cingulate cortex. In the proposed circuit, these two regions are treated as complementary communication stations. The fronto-insular cortex acts as a deep collecting and broadcasting station, whereas the anterior cingulate cortex acts as the receiving and integrative station. Accordingly, Raslan Circuit proposes that von Economo neurons are not merely histological markers of higher cognition. They are the cellular antenna elements of the circuit. Through their elongated dipole-like structure and their selective presence in the FI–ACC axis, VENs may provide a mechanism by which integrated information is transformed into a transmissible electromagnetic signal and then received by a complementary cortical station.

This does not mean that conventional synaptic transmission is rejected. The proposed circuit is complementary. Conventional axonal and synaptic communication remains essential for neural signaling, but the unique morphology and position of VENs may allow them to participate in an additional electromagnetic mode of information organization, tuning, transmission, and reception.

### 2.4 The FI–ACC axis as a paired biological antenna system

The FI–ACC axis is proposed as a paired biological antenna system in which both stations participate in bidirectional, synchronously coupled electromagnetic communication, rather than a strictly unidirectional transmitter–receiver architecture. Although FI and ACC occupy asymmetric functional positions within the circuit—FI serving as a deep multimodal collecting and organizing station, and ACC serving as a receiving and redistributive hub—this functional asymmetry does not preclude reciprocal field coupling. A dipole antenna element, by physical definition, is capable of both transmitting and receiving electromagnetic signals. Accordingly, VENs in both FI and ACC are proposed to participate in bidirectional field-sensitive coupling, in which each population can simultaneously organize, transmit, and receive field-based information along the FI–ACC axis.

FI occupies the functionally organizing pole of the circuit: it is located near the convergence of sensory, interoceptive, autonomic, emotional, and cognitive streams around the lateral sulcus, and its VENs are proposed to assemble and format multimodally integrated signals. ACC occupies the functionally redistributive pole: it is embedded in networks for salience detection, attention, working memory, decision-making, action selection, and thalamocortical redistribution, and its VENs are proposed to receive, evaluate, and route the incoming signal toward these downstream systems. This functional asymmetry—collecting and organizing in FI, receiving and redistributing in ACC—coexists with bidirectional electromagnetic coupling, such that both poles of the circuit participate in resonance-sensitive field communication simultaneously.

This subsection defines only the anatomical logic of the paired system. The detailed role of ACC as a receiving and redistributive hub is developed later, after the electromagnetic rationale and functional consciousness framework have been established.

## 3 Electromagnetic and biophysical rationale

### 3.1 The dipole distinction: electric dipole versus dipole antenna

Before evaluating the relationship between Raslan Circuit and existing electromagnetic accounts of consciousness, it is necessary to distinguish between two different meanings of the term dipole. In physics and neuroscience, a dipole often refers to charge separation or to a neural current-dipole model used to describe local field generation. In antenna theory, by contrast, a dipole refers to a structure whose geometry supports resonance, transmission, and reception.

A dipole is generally defined as a system with two opposite poles, charges, or polarities separated by a distance. However, the same term has different meanings in different scientific contexts. This distinction is essential for understanding how Raslan Circuit differs from existing electromagnetic field theories of consciousness.

In electrostatics, an electric dipole refers to two opposite charges separated in space. In neuroscience, neural current dipoles are often used to model local electrical fields generated by aligned neuronal populations, particularly pyramidal neurons. These dipoles help explain local field potentials, EEG, MEG, and extracellular field patterns. Their primary role is descriptive and field-generating: they model how local neural activity produces measurable electrical and magnetic fields.

A dipole antenna, by contrast, is not merely a charge distribution. It is a communication device. A classical antenna dipole consists of two aligned conductive elements separated by a feed point. When oscillating electrical activity is applied, the antenna converts electrical energy into electromagnetic radiation; when exposed to an incoming electromagnetic wave, it can convert that wave back into electrical activity. Its key concepts are resonance, wavelength, impedance, tuning, radiation pattern, transmission, and reception.

Raslan Circuit relies on the second meaning of dipole. It is not primarily a theory of static charge separation or local field generation. It proposes that VEN morphology provides a biological dipole antenna architecture capable of tuning, transmitting, and receiving electromagnetic information between FI and ACC.

### 3.2 Relationship to electromagnetic field theories of consciousness

Clarifying the dipole distinction also helps define the relationship between Raslan Circuit and electromagnetic field theories of consciousness. The two approaches overlap in taking endogenous electromagnetic activity seriously, but they differ in explanatory target, anatomical emphasis, and proposed mechanism. Whereas EMF theories primarily focus on the field configurations associated with conscious states, Raslan Circuit focuses on a candidate cellular substrate and anatomical pathway through which integrated information may be transmitted toward anterior conscious-access systems.

This distinction is central to the present hypothesis. Raslan Circuit does not deny that neurons generate local electromagnetic fields in the conventional current-dipole sense. Rather, it proposes that the elongated morphology and selective anatomical distribution of VENs justify a second, communication-oriented interpretation in which a specialized neuronal population may support resonance-sensitive transmission and reception between FI and ACC.

Electromagnetic field theories of consciousness propose that conscious experience is related to specific electromagnetic field patterns generated by brain activity. One influential formulation emphasizes local field potentials and spatial electromagnetic configurations produced by synchronous synaptic activity and aligned cortical neurons (Pockett, 2012). However, Raslan Circuit approaches the problem from a different direction. Existing EMF theories often focus on local field patterns generated by neural current dipoles, especially in relation to pyramidal neuronal architecture and local cortical layering. Raslan Circuit instead proposes a communication-oriented model: a specific FI–ACC–VEN pathway in which specialized biological antenna elements organize, transmit, receive, and redistribute information.

Thus, Raslan Circuit does not reject electromagnetic theories of consciousness. It adds an anatomical and cellular substrate that many field-based theories lack. The present model therefore shifts the emphasis from field pattern alone to the specialized circuit that may organize and convey such information.

In this respect, Raslan Circuit is best understood as a member of the broader family of electromagnetic field (EMF) theories of consciousness, rather than as a competing alternative. It preserves the core insight that consciousness is closely linked to structured electromagnetic activity in the brain, while specifying a particular anatomical and cellular substrate—the FI–ACC–VEN axis—through which such field dynamics may be organized and functionally exploited. Hales (2014) and subsequent EMF accounts have argued that the brain’s endogenous electromagnetic field can contribute directly to conscious experience, and Hunt and Jones (2023) have contrasted such field-based hypotheses with purely spike-based coding views. Raslan Circuit aligns with these proposals by treating field organization as functionally significant, but it adds a concrete, testable anatomical implementation in which von Economo neurons are posited to serve as elongated, field-sensitive elements that help shape and transmit consciously relevant patterns along the FI–ACC axis.

### 3.3 Ephaptic coupling as the physical foundation

Ephaptic coupling refers to the influence of extracellular electric fields on neuronal membrane potentials independent of direct synaptic transmission. Neural activity produces transmembrane currents, ionic fluxes, extracellular potentials, and local field gradients. Under certain conditions, these fields can influence spike timing, synchronize neuronal populations, and modulate network dynamics (Richardson et al., 1984; Frohlich and McCormick, 2010; Anastassiou et al., 2011; Buzsáki et al., 2012; Chiang et al., 2019; Hunt and MacIver, 2026).

The ephaptic coupling literature is important for Raslan Circuit because it establishes a general physical principle: neuronal electromagnetic fields can affect neuronal function. This does not prove Raslan Circuit, but it supports the plausibility of the basic premise that field-based communication can contribute to neural information processing.

Classical ephaptic coupling is usually discussed as a local or near-field mechanism. Raslan Circuit extends this principle by proposing a more specialized anatomical system in which VENs act as biological antenna elements. In this model, VENs do not merely experience passive field effects; they organize, transmit, receive, and redistribute field-based information within a defined FI–ACC circuit.

### 3.4 From ephaptic fields to antenna-based communication

The key conceptual transition in Raslan Circuit is from local field interaction to organized antenna-based communication. In conventional ephaptic coupling, the field is often treated as a local influence surrounding active neural tissue. In the present framework, the field is interpreted as a potential communication medium shaped by specialized cellular geometry (see Table 1).

**Table 1:** Conceptual distinction between electric dipoles and dipole antennas.

Aspect	Electric dipole	Dipole antenna
Basic meaning	Two opposite charges or poles separated by distance	Two conductive arms separated by a feed point and configured for transmission or reception
Scientific context	Electrostatics, polarization, and neural current modeling	Electromagnetics, communications, and antenna theory
Main role	Describes charge separation or local field generation	Supports tuning, transmission, reception, and resonance
Typical neural relevance	Neural current dipoles underlying LFP, EEG, and MEG interpretations	Conceptual model for a communication-oriented biological structure
Radiation function	Not inherently a radiating system	Specifically analyzed as a transmitting or receiving system
Relevance to Raslan Circuit	Helps explain field generation	Provides the operative analogy for VEN-based communication

A biological dipole antenna does not need to behave identically to a metallic antenna in free space. The brain is a conductive, hydrated, lossy, and dispersive biological medium. Therefore, the proposed antenna function of VENs should be understood within the constraints of neural tissue. The expected mechanism may involve near-field coupling, resonance, dielectric boundary effects, tissue-specific field shaping, volume conduction, and phase coherence rather than simple free-space radiation.

However, the analogy to antenna physics remains central. The elongated bipolar-like morphology of VENs may allow them to align with field gradients, support directional coupling, and participate in resonance-based communication. Their paired distribution in FI and ACC provides an anatomical basis for a bidirectionally coupled biological antenna system (see Table 2).

**Table 2:** Raslan Circuit compared with electromagnetic field theories of consciousness.

Aspect	EMF theories of consciousness	Raslan Circuit
Basic anatomical unit	Aligned cortical neurons, especially pyramidal populations	Von Economo neurons in FI and ACC
Main EM emphasis	Local field configurations associated with conscious states	Communication-oriented coupling across a specific cortical pathway
Dipole interpretation	Neural current dipole	Antenna-like dipole analogy
Primary role	Correlating field pattern with conscious experience	Organizing, transmitting, receiving, and redistributing integrated information
Spatial emphasis	Local or regional cortical field architecture	Long-range FI–ACC communication axis
Mechanistic novelty	Consciousness linked to specific EM field configurations	Specialized cellular substrate for field-based integration

### 3.5 Spectral prediction: the terahertz hypothesis

If VENs are interpreted as biological dipole antenna elements, their physical dimensions permit a preliminary resonance estimate based on classical electromagnetic relations. Under a simplified half-wave dipole assumption, the effective resonant wavelength can be approximated as *λ* ≈ 2 *L*, where *L* is the effective dipole length, and the corresponding frequency can be estimated as *f* = *v*/*λ*. In free space, *v* is approximated by *c*; in biological tissue, however, v is reduced by dielectric properties and the estimates must therefore be treated as heuristic rather than measured biological frequencies. These assumptions are summarized before Table 3 to make the spectral prediction explicit and falsifiable rather than definitive.

**Table 3:** Heuristic VEN-scale resonance estimates under simplified dipole assumptions.

VEN scale	Approximate dipole length	Approximate resonant wavelength	Estimated frequency range	Band
Small VEN-scale span	1,500–2,500 μm	3,000–5,000 μm	60–100 THz	Far infrared/terahertz
Large VEN-scale span	5,000–6,000 μm	10,000–12,000 μm	25–30 THz	Terahertz

The values in Table 3 are therefore not presented as established physiological frequencies. They are a constrained theoretical estimate showing that VEN-scale dimensions, if treated through a simplified dipole-antenna framework, point toward very high-frequency electromagnetic ranges, potentially including terahertz or far-infrared domains. Because neural tissue is hydrated, conductive, lossy, and dispersive, any real biological mechanism would be expected to involve near-field coupling, tissue-specific dielectric boundaries, and resonance-sensitive field organization rather than simple free-space radiation.

The value of the terahertz hypothesis is not that it proves the mechanism, but that it makes the Raslan Circuit falsifiable. If VEN-rich FI and ACC tissue has distinctive electromagnetic resonance or field-sensitivity properties, future terahertz spectroscopy, near-field imaging, computational electromagnetic modeling, or laminar electrophysiology may detect measurable differences between VEN-rich and VEN-sparse cortical regions.

## 4 Raslan Circuit and functional consciousness

Raslan Circuit is not advanced as a complete theory of consciousness, but as a proposed anatomical and biophysical mechanism that may help implement functions described by several existing frameworks. The anatomical and biophysical arguments developed so far support a specific claim: the fronto-insular cortex (FI), the anterior cingulate cortex (ACC), and their von Economo neurons (VENs) form a selectively specialized axis with plausible field-sensitive properties. The present section addresses how such an axis may contribute to functional consciousness, understood as the set of processes by which information becomes consciously accessible, usable for goal-directed behavior, and integrated over time.

Within the embedded-processes view, Cowan et al. (2025) proposed that conscious contents correspond to a limited-capacity focus of attention, with the intraparietal sulcus (IPS) acting as a hub through which attended information is funneled from posterior representational systems. The IPS is strongly connected to posterior sensory and association cortices and plays a key role in determining which information enters and remains in the focus of attention. At the same time, this view emphasizes that frontal regions are more closely linked to the control of attention and action than to the sensory substrate of awareness itself. Within this architecture, Raslan Circuit is best understood as complementary. The IPS hub is proposed as a parietal focus that indexes and coordinates conscious contents, whereas the FI–ACC–VEN axis is proposed as a deep paralimbic pathway that assembles interoceptively grounded, salience-weighted, multimodal information in FI and conveys it to ACC and anterior control systems. In short, the IPS may specify which representations are in the focus of attention; Raslan Circuit proposes one way in which certain behaviorally significant representations are prepared and delivered to the anterior systems that act on them.

A similar complementarity holds with the awareness-buffer reformulation of the multicomponent working memory model. Allen et al. (2026) reconceptualized the episodic buffer as an awareness buffer: a limited-capacity, multimodal, consciously accessible integration space whose contents correspond closely to the current focus of awareness and are shaped by both top-down control and bottom-up salience. The awareness buffer aggregates perceptual input, internally generated thought, long-term memory, and bodily states into a unified internal model available for reasoning, report, and action. Raslan Circuit can be interpreted as a candidate input–output pathway to this buffer. FI, with its VEN-rich architecture and deep convergence of sensory, interoceptive, and affective signals, is a plausible site at which awareness-buffer-relevant content is assembled; ACC, also VEN-rich and strongly connected to executive, motor, limbic, and thalamic systems, is a plausible site at which that content is received, weighted, and redistributed. On this reading, Raslan Circuit does not replace the awareness buffer as a cognitive construct; it provides a concrete anatomical and biophysical route through which some of the buffer’s contents may be constructed and delivered.

Global Workspace Theory offers a broader architectural account, in which conscious access corresponds to ignition and widespread broadcast within a dynamic cortico-thalamic workspace. Recent formulations of this framework emphasize that prefrontal and posterior regions jointly participate in flexible global broadcasting, and that conscious events often integrate sensory, interoceptive, emotional, and metacognitive components. Raslan Circuit is consistent with this picture but operates at a more localized level. Where Global Workspace Theory describes the global consequence of ignition—widespread availability—Raslan Circuit addresses a specific route by which multimodally integrated, salience- and body-weighted information may reach the anterior systems that participate in such ignition. FI is proposed to organize and format this information; ACC, with its extensive cortical and thalamic projections, is proposed to inject it into the broader workspace. In this way, the FI–ACC–VEN axis may contribute to the upstream phase that prepares content for global broadcast, rather than constituting the workspace itself.

Information Integration Theory adds a complementary perspective by formalizing consciousness as integrated information within a system that generates more information as a whole than the sum of its parts. Contemporary work in this tradition emphasizes the thalamocortical complex and recurrent connectivity as key to such integration. Raslan Circuit aligns with this framework in two ways. First, it offers a concrete example of how differentiated information from multiple posterior and bodily sources might be integrated along a specific anatomical route before contributing to larger thalamocortical complexes. Second, ACC’s dense reciprocal connections with mediodorsal and anterior thalamic nuclei provide a natural interface through which FI–ACC activity could influence the broader integrated causal structure emphasized by this theory. Thus, the theory explains why integration is necessary for consciousness; Raslan Circuit proposes one anatomically and cellularly specific way such integration may be instantiated for a particular class of content.

Taken together, these convergences suggest a consistent functional interpretation. Embedded-processes models and the awareness-buffer framework identify a capacity-limited, multimodal, consciously accessible workspace; Global Workspace Theory characterizes how contents of that workspace become globally available through ignition and broadcast; Information Integration Theory characterizes why integrated causal structure matters for conscious experience. Raslan Circuit proposes that the FI–ACC–VEN axis may serve as a specialized communication infrastructure that feeds these more global mechanisms, particularly for information that is multimodally synthesized, closely tied to interoceptive and affective state, and behaviorally salient. The circuit is therefore advanced not as a rival theory of consciousness, but as a bridging hypothesis linking the anatomical uniqueness of VENs and the biophysical plausibility of field-sensitive coupling to established functional accounts of conscious access.

## 5 ACC as a receiving and redistributive hub

The anterior cingulate cortex occupies a strategic position at the interface of salience detection, attentional control, emotional valuation, conflict monitoring, effort allocation, decision-making, and action selection. It receives convergent inputs from frontal, limbic, thalamic, and insular systems and projects broadly to motor, autonomic, and executive networks. Within Raslan Circuit, this anatomical position makes ACC the primary redistributive station within a bidirectionally coupled FI–ACC axis: the site at which VEN-mediated, multimodally integrated, interoceptively grounded signals—organized through reciprocal field coupling between FI and ACC—are transformed into context-sensitive patterns of activity that influence conscious access and behavior.

The VEN population in layer Vb of ACC is central to this interpretation. VENs in ACC are not simply a duplicate of VENs in FI; they occupy the redistributive pole of the proposed FI–ACC axis and are embedded in downstream circuitry that includes executive, limbic, motor, and autonomic targets. Their elongated, bipolar morphology mirrors that of VENs in FI, supporting the idea that they are tuned to the same type of oriented field organization, yet their network context is different: where FI VENs are positioned to assemble, organize, and participate in bidirectional field coupling, ACC VENs are positioned to evaluate, redistribute, and equally participate in reciprocal coupling back toward FI. On this view, VENs in ACC serve as the cellular elements through which field-organized signals exchanged bidirectionally along the FI–ACC axis are converted into new patterns of activity that can be routed selectively toward multiple output systems.

This redistributive role allows ACC to integrate signals arriving through the bidirectional FI–ACC coupling with ongoing task demands, motivational state, and top-down control. The output of ACC is therefore not a simple copy of FI activity; it is a contextually reweighted version of that signal, shaped by current goals, internal state, and environmental contingencies. Functionally, this makes ACC a plausible anatomical substrate for bridging between the VEN-based bidirectional communication mechanism proposed in Raslan Circuit and the broader conscious-access architectures described by existing theories. For example, in the embedded-processes and awareness-buffer frameworks, ACC can be seen as one of the anterior systems that shape which FI–ACC-coupled contents are granted sustained access to the focus of attention and to the consciously accessible awareness buffer. In Global Workspace Theory, the same properties make ACC a natural contributor to ignition and broadcast, especially for contents that are tightly coupled to salience, effort, and action preparation.

ACC’s dense reciprocal connectivity with mediodorsal and anterior thalamic nuclei further extends its redistributive role. Through these thalamocortical loops, activity received and transformed in ACC can be propagated to a wide range of cortical territories beyond those reachable by direct corticocortical projections alone. In the context of Information Integration Theory, this thalamic interface allows Raslan Circuit to feed into the integrated causal structures that the theory associates with conscious experience. The FI–ACC–VEN axis would then be one of the pathways through which differentiated posterior and bodily information is assembled, weighted, and injected into the thalamocortical complex that supports globally integrated conscious states.

Together, these considerations support the operational role assigned to ACC in the model: it receives and reciprocally exchanges VEN-mediated signals with FI through bidirectional field coupling, reweights incoming information according to task demands and internal state, and redistributes it through executive, limbic, motor, autonomic, and thalamocortical systems. This makes ACC the final anatomical and functional bridge between the proposed VEN-based communication mechanism and large-scale processes associated with conscious access and control.

## 6 Testable predictions and empirical approaches

Any hypothesis about consciousness must ultimately be judged by the clarity and falsifiability of its predictions. Raslan Circuit is formulated with this requirement in mind. Its specificity at the anatomical level (FI and ACC), the cellular level (VENs in layer Vb), and the biophysical level (field-sensitive, antenna-inspired coupling) allows a series of converging empirical tests, ranging from connectivity analyses to clinical studies and computational modeling.

### 6.1 Anatomical and functional connectivity predictions

If Raslan Circuit is correct, FI and ACC should show preferential structural and functional coupling relative to other insular–cingulate pairings. High-resolution diffusion MRI and tractography should reveal robust white-matter pathways linking VEN-rich territories in FI and ACC, with a connectivity profile stronger or more specialized than that between FI and VEN-poor cingulate regions. Functionally, task-based and resting-state fMRI should show that FI and ACC coactivate and exhibit enhanced functional connectivity during conditions requiring multimodal integration, interoceptive awareness, salience evaluation, or affectively weighted decision-making. Disruption of this pathway—by focal lesions, targeted neurosurgical intervention near VEN-rich FI or ACC territories, or non-invasive neuromodulation focused on these regions—should selectively impair the integration of interoceptive and affective context into conscious access, while sparing basic posterior sensory processing.

### 6.2 Cellular, laminar, and field-level predictions

At the cellular and laminar level, the hypothesis predicts that VEN-rich layer Vb in FI and ACC will exhibit distinctive field organization relative to neighboring pyramidal-dominated layers. Laminar current-source-density analyses and high-density intracranial recordings should reveal oriented, depth-spanning field components associated with VEN populations during tasks that recruit multimodal integration and salience processing. These components should show stronger coupling between FI and ACC than between FI and VEN-sparse regions, and their disruption should correlate with changes in timing, phase relationships, or synchrony of activity across the FI–ACC axis.

At a more speculative but distinctive level, the resonance-based extension of Raslan Circuit predicts that VEN-dense tissue in FI and ACC may display selective spectral properties—including differential sensitivity or response—when probed with high-frequency electromagnetic fields. Terahertz and near-infrared spectroscopy applied to postmortem tissue from VEN-rich versus VEN-poor regions, combined with finite-element electromagnetic modeling of VEN-like geometries, could test whether VEN architecture supports unusual frequency-selective coupling properties. Even if such experiments ultimately falsify the terahertz-specific prediction, they would still provide informative constraints on the biophysical limits of the antenna-inspired interpretation. The more speculative open-ended antenna-configuration ideas should be treated, if explored at all, as downstream experimental extensions rather than as core claims of the present model.

### 6.3 Predictions for conscious access and clinical states

If Raslan Circuit contributes to the transition from distributed representation to conscious access, then selective disruption of the FI–ACC–VEN axis should produce specific alterations in conscious experience. Patients with focal damage to VEN-rich FI or ACC, or conditions known to reduce VEN density—such as behavioral-variant frontotemporal dementia—should show disproportionate impairments in salience-based conscious access, emotional awareness, interoceptive precision, and the affective coloring of conscious content, even when posterior sensory function remains relatively preserved. Conversely, states associated with abnormal hyperactivation of insula–ACC circuits—such as certain forms of panic, pain catastrophizing, or particular epileptic auras—may reveal exaggerated or dysregulated contributions of the proposed circuit to conscious experience. These clinically testable predictions connect Raslan Circuit to the clinical neuroscience of awareness, allowing the hypothesis to be tested against patterns of deficit and excess in real patients.

### 6.4 Computational modeling and integrative tests

A complementary line of testing involves computational modeling of VEN-based field interactions in FI and ACC. Treating VENs as elongated, bipolar elements embedded in a conductive, dispersive medium, finite-element electromagnetic simulations can generate quantitative predictions about field geometry, coupling strength, and resonance behavior under plausible physiological conditions. These predictions can then be compared with empirical measurements of extracellular field distributions and phase relationships in FI and ACC, providing a bridge between the antenna-inspired modeling and *in vivo* electrophysiology.

Crucially, none of these tests needs to be performed in isolation. The strength of Raslan Circuit as a scientific proposal lies not in any single spectacular prediction, but in the pattern of results that would emerge if its anatomical, cellular, biophysical, and clinical expectations converge or diverge together. Convergence across structural connectivity, field measurements, computational models, and clinical syndromes would support the view that the FI–ACC–VEN axis plays a distinctive role in multimodal integration and conscious access; systematic failure across these domains would argue against the hypothesis and help refine or replace it. Either outcome, positive or negative, would move the conversation about consciousness from abstract speculation toward experimentally grounded mechanisms.

## 7 Conclusion

The Raslan Circuit is proposed as a testable bridging hypothesis linking the fronto-insular cortex, the anterior cingulate cortex, and von Economo neurons to functional models of conscious access. The model provides a specific anatomical and biophysical framework through which multimodal and salience-weighted information may be integrated and conveyed toward systems involved in awareness, working memory, and behavioral control. Its value therefore lies not in replacing existing theories of consciousness, but in offering a mechanistically explicit pathway that can be examined empirically.

## Data Availability

The original contributions presented in the study are included in the article/Supplementary material, further inquiries can be directed to the corresponding author.
