# Supplementary material for: Raslan Circuit: von Economo neurons as biological dipole antenna elements in a proposed FI–ACC communication pathway for multimodal integration and conscious access
Source: Front Syst Neurosci. 2026 Aug 21;20:1779601. doi: 10.3389/fnsys.2026.1779601 (PMC13541754; doi:10.3389/fnsys.2026.1779601)
Supplement: Supplementary file 1 [file Data_sheet_1.pdf]

Supplementary Historical Appendix: Foundational Developments Underlying the Raslan Circuit Hypothesis

Submitted as Optional Supplementary Material
Raslan Circuit: Von Economo Neurons as Biological Dipole Antenna Elements in a Proposed FI–ACC Communication Pathway for Multimodal Integration and Conscious Access
Frontiers in Systems Neuroscience — Major Revision

Preface

This appendix provides historical context for three foundational threads that converge in the Raslan Circuit hypothesis: the discovery of electromagnetic waves and Maxwell's unified field theory; the emergence of antenna theory from Hertz's laboratory experiments to modern antenna engineering; and the discovery and progressive characterization of von Economo neurons from the 19th century to the present day. The material assembled here was condensed or omitted from the main manuscript in the interest of conciseness, but may assist readers who benefit from situating the hypothesis within its broader intellectual lineage. The editor and reviewers are invited to include or exclude this appendix at their discretion.

Part I: The Discovery of Electromagnetic Waves — From Ørsted to Maxwell to Hertz

I.1 The Age of Separation: Electricity and Magnetism as Distinct Forces

For most of human history, electricity and magnetism were treated as entirely independent natural phenomena. Amber's ability to attract light materials when rubbed, and the compass needle's alignment with the Earth's poles, were catalogued as separate curiosities without any suspected connection. This conceptual isolation persisted well into the early modern period, with electricity studied through friction machines and Leyden jars, while magnetism remained a navigational tool with poorly understood physical principles.

I.2 Ørsted and Faraday: The Hidden Dance Revealed

The first decisive crack in this separation came in 1820, when the Danish physicist Hans Christian Ørsted made a serendipitous observation: an electric current flowing through a wire could deflect a nearby compass needle. This discovery—that electricity could produce magnetic effects—was paradigm-shifting. It demonstrated that the two forces were not merely analogous but physically coupled. Within eleven years, Michael Faraday extended this symmetry dramatically. In 1831, Faraday showed that a *changing* magnetic field could induce an electric current in a nearby conductor—the phenomenon now known as electromagnetic induction. Together, Ørsted and Faraday revealed a reciprocal, dynamic relationship: moving charges

create magnetic fields; changing magnetic fields create electric currents. The stage was set for a unified mathematical account.[1][2]

I.3 Maxwell's Synthesis: The Most Spectacular Triumph of Theoretical Physics

The full unification of electricity, magnetism, and light was achieved not in a laboratory but on paper. Between 1860 and 1871, James Clerk Maxwell, working at King's College London and at his family home Glenlair, developed a unified field theory that synthesized all known electromagnetic phenomena into a coherent mathematical framework. His two most important papers were "On Physical Lines of Force" (1861), which introduced the concept of displacement current as a correction to Ampère's circuital law, and "A Dynamical Theory of the Electromagnetic Field" (1865), in which he derived the electromagnetic wave equation.[3][2][^1]

The decisive insight was quantitative. When Maxwell calculated the theoretical velocity of the self-sustaining electromagnetic wave predicted by his equations, it matched the known speed of light with extraordinary precision. His conclusion was as radical as it was elegant: light itself is an electromagnetic wave. This pronouncement—that a purely mechanical phenomenon (light) and a purely electrical phenomenon (electromagnetic radiation) were manifestations of the same underlying physics—has been called "the most stunning triumph of theoretical physics in the 19th century". Maxwell's equations today underpin all modern information and communication technologies.[4][2]

I.4 Hertz: From Mathematical Prophecy to Physical Reality

Maxwell's prediction remained theoretical for over two decades. It was Heinrich Hertz who, in 1887–1888, provided definitive experimental confirmation. Using a spark-gap transmitter—a center-fed dipole constructed from two collinear copper rods with metal spheres at their ends—Hertz generated invisible electromagnetic waves and detected them with a resonant loop antenna placed several meters away. When a spark was generated at the transmitter, a corresponding spark appeared at the receiver gap almost simultaneously: electromagnetic energy had propagated wirelessly through space.[5][6][7][8][^9]

Hertz went further, demonstrating that these waves could be reflected, refracted, and polarized—exactly as Maxwell's theory predicted for light. Ironically, Hertz famously doubted the practical utility of his discovery. He reportedly declared it "of no use whatsoever." History proved otherwise. The Hertzian dipole—named in his honour—became the prototype for all antenna-based communication systems that followed, and its physics remain central to the antenna-inspired interpretation of von Economo neuron morphology advanced in Raslan Circuit.[^10]

Part II: The Genesis and Evolution of Antenna Theory

II.1 Hertz's Accidental Invention: The World's First Dipole Antenna

Paradoxically, the history of the antenna begins before the device even had a name. Hertz's 1887 transmitter was, in retrospect, a half-wave dipole antenna: two horizontal conducting rods with metal spheres creating capacitive loading at the ends, with a spark gap at the center. The receiving apparatus was a resonant loop—the world's first receiving antenna. These structures allowed Hertz to demonstrate that electromagnetic energy could be intentionally radiated into free space and captured at a distance, establishing the fundamental transmitter–receiver architecture that Raslan Circuit invokes as a biological analogy.[6][7][^9]

The operating frequency of Hertz's early experiments was in the range of 50–450 MHz, determined by the physical length of his dipole elements. This frequency–length relationship—in which the resonant frequency of a half-wave dipole is inversely proportional to its physical length—is the same mathematical relationship applied in the Raslan Circuit hypothesis to estimate the putative resonant properties of von Economo neurons from their known physical dimensions.[^6]

II.2 Marconi and the Monopole: From Room to Globe

Hertz's apparatus could transmit signals only across the span of a single room. The transition to global communication was catalyzed by Guglielmo Marconi in the mid-1890s. Recognizing that transmission range was fundamentally limited by the physical scale of the radiating structure, Marconi made a groundbreaking empirical discovery: by connecting one terminal of his transmitter to a long vertical wire elevated high in the air and the other terminal to the earth, he dramatically increased both the effective wavelength and the radiation efficiency.[11][12]

On 12 December 1901, from Signal Hill in Newfoundland, Marconi and his assistant George Kemp confirmed reception of the first transatlantic wireless signal—Morse code for the letter "S"—transmitted from Poldhu, Cornwall, across 2,100 miles of open ocean. The transmitting antenna at Poldhu consisted of fifty wires suspended in a fan shape from two 160-foot poles; the receiving antenna in Newfoundland was a 400-foot wire kept aloft by a kite. This demonstration established that radio signals could follow the curvature of the Earth, giving wireless communication a genuinely global dimension and transforming Hertz's laboratory curiosity into the foundation of the modern communications age.[13][14][12][11]

II.3 Yagi and Uda: Directional Gain and Beam Steering

The early 20th century brought a crucial insight: antennas need not radiate omnidirectionally. Beginning in 1924 at Tohoku Imperial University in Japan, Shintaro Uda and Hidetsugu Yagi designed and constructed a highly directional antenna by arranging passive parasitic elements—reflectors and directors—alongside a driven dipole. The first detailed English-language description of this Yagi-Uda antenna was published in 1926.[15][16]

The key innovation was that passive conducting elements, placed at carefully calculated spacings and lengths relative to the driven element, could redirect and concentrate the radiated energy into a narrow forward beam. Reflectors, positioned behind the driven element and slightly longer, provided inductive reactance that reinforced the forward radiation; directors, placed in front and slightly shorter, provided capacitive reactance that further

focused the beam. The result was a high-gain, directional antenna with a unidirectional beam pattern—a principle that would prove transformative for radar, television, satellite communication, and modern wireless networks.[17][18][16][15]

The Yagi-Uda antenna is directly relevant to the conceptual architecture of Raslan Circuit. The fronto-insular cortex and the anterior cingulate cortex, each equipped with a specialized population of elongated, bipolar von Economo neurons, may be understood as occupying the transmitter and receiver positions of a biological antenna pair—analogous to the way that the driven element and receiving structure of a Hertzian dipole system define complementary communication roles.

II.4 From Metallic Rods to Intelligent Arrays: The Modern Antenna Landscape

The second half of the 20th century witnessed rapid diversification in antenna design driven by the demands of radar, television, satellite communication, and eventually mobile telephony. Frequency-independent designs, including log-periodic and horn antennas, accommodated the ultra-high frequencies required for satellite and deep-space applications. The miniaturization demands of the silicon age gave rise to microstrip patch antennas—thin, planar elements etched onto printed circuit boards—enabling antennas to be embedded invisibly into consumer electronics.[^7]

Contemporary antenna technology is characterized by dynamic, software-driven spatial multiplexing. In MIMO (Multiple-Input Multiple-Output) systems and phased array architectures, antennas no longer radiate statically; instead, intelligent algorithms steer precise beams toward individual users, maximizing spectral efficiency in real time. This trajectory—from Hertz's static bench-top dipole to dynamic, context-sensitive beam forming—illustrates how the fundamental physics of dipole radiation established in 1887 underlies a continuously expanding repertoire of communication architectures.

Part III: The Discovery and Characterization of Von Economo Neurons

III.1 Pre-History: Early Observations Before von Economo

The neurons now named after Constantin von Economo were not entirely unnoticed before his systematic description. Vladimir Betz, the Ukrainian anatomist known for identifying the large pyramidal neurons of the motor cortex that also bear his name, observed unusually large cells in the cingulate gyrus as early as 1874–1881 and remarked on their exceptional size relative to neighbouring layer V neurons. In 1895, Carl Hammarberg noted similar cells in what he called the "Gyrus Centralis Anterior," a region corresponding in contemporary nomenclature to the precentral gyrus. Santiago Ramón y Cajal, arguably the greatest neuroanatomist in history, highlighted the prevalence of large, spindle-like cells in the infragranular layers of the cingulate gyrus and the insula in 1899–1901. Rose (1927) subsequently described comparable cells in the anterior cingulate cortex of chimpanzee brain. Despite these scattered observations, no systematic morphological characterization or anatomical mapping was attempted, and the cells fell outside the mainstream focus of early 20th-century neuroanatomy.[19][20]

III.2 Von Economo's Definitive Description: 1925–1926

The first comprehensive and rigorous characterization of these neurons was provided by Constantin von Economo (1876–1931), an Austrian psychiatrist and neurologist, in his landmark cytoarchitectonic study of the human cerebral cortex conducted with Georg N. Koskinas. In 1925, von Economo and Koskinas published their exhaustive cortical atlas using Nissl staining, confirming the presence of a highly distinctive population of large, elongated neurons predominantly located in layer Vb of two specific cortical regions: the fronto-insular cortex (FI) and the anterior cingulate cortex (ACC).[21][20][22][23]

Von Economo (1926) described these cells as "rod cells" and "corkscrew cells," referring to their straight and twisted morphological variants respectively. Their unusual stick-like or corkscrew-like soma was so striking that von Economo initially entertained the possibility that they represented a pathological alteration, before recognizing them as a specialized neuronal subtype. He also explicitly distinguished these specialized cells from the more common fusiform or spindle-shaped neurons found throughout the cerebral cortex—a distinction that remains important for the Raslan Circuit hypothesis, which is anchored specifically in the FI–ACC neuronal population rather than in elongated neurons generally.[20][22][^19]

III.3 The Silent Period and Rediscovery: 1930s–1999

Following von Economo's original description, a prolonged period of relative scientific neglect ensued. Ngowyang (1936) reported the presence of similar neurons in the subiculum and entorhinal cortex, extending the anatomical picture somewhat. However, for most of the mid-20th century, VENs fell outside the focus of mainstream neuroscience, overshadowed by the rapid advances in synaptic physiology, molecular neurobiology, and neuroimaging that characterized the postwar decades.[^19]

The rediscovery of VENs was initiated in the 1990s by Esther Nimchinsky and colleagues, who provided the first modern quantitative and qualitative description of these cells using contemporary histological and immunohistochemical methods. Nimchinsky et al. (1995) and their follow-up work in 1999 established rigorous anatomical and morphological criteria for VEN identification and confirmed their selective concentration in layer Vb of ACC and FI in great apes and humans. This rediscovery opened the contemporary era of VEN research.[24][25][^19]

III.4 Allman and Colleagues: Functional Hypotheses and Evolutionary Perspective

A major conceptual breakthrough came in 1999 when John Allman and colleagues at the California Institute of Technology published the first report specifically linking VENs to questions of consciousness, social cognition, and self-awareness. They noted the striking selective presence of VENs in hominids but not in other, non-great-ape primate species, suggesting that VENs represented a phylogenetic specialization associated with the expansion of complex social cognition in the great ape lineage.[25][21][^24]

In 2003, at a Society for Neuroscience meeting, Allman reported on VENs in the fronto-insular cortex specifically—a region that had undergone significant evolutionary adaptations in humans, perhaps as recently as 100,000 years ago. The large size and simple dendritic structure

of VENs led Allman and colleagues to propose that these neurons might function as rapid-projection neurons, sending basic but critical information from FI and ACC to other brain regions at high speed, while slower neighbouring pyramidal neurons carried more detailed information. This fast-relay hypothesis is conceptually consistent with—though mechanistically distinct from—the field-based communication role proposed for VENs in Raslan Circuit.[21][25]

III.5 The Evolutionary Distribution: Convergent Emergence Across Large-Brained Species

One of the most biologically significant findings in VEN research has been the discovery that these neurons are not unique to humans or even to primates. Hof and Van der Gucht (2007) reported the presence of VENs in humpback, fin, sperm, and killer whales. Subsequent work by Butti et al. (2009) extended this to additional cetacean species including the bottlenose dolphin, Risso's dolphin, and the beluga whale, with VEN distributions in these species comparable to those reported in humans and great apes. Hakeem et al. (2009) confirmed the presence of VENs in African and Indian elephants, where they are most abundant in the frontoinsular cortex and also present in the anterior cingulate and certain prefrontal regions.[26][27][^28]

The phylogenetic distribution of VENs—present in hominids, cetaceans, and elephants, but absent in most other mammals including non-great-ape primates—points toward convergent evolution under comparable selective pressures. All three groups share very large absolute brain sizes, highly complex social structures, evidence of self-awareness, and sophisticated communicative repertoires. This convergent pattern supports the hypothesis that VENs represent a functional specialization for rapid, long-range integration of social, emotional, and interoceptive information in brains that have reached a threshold of size and complexity—precisely the functional role proposed for them in Raslan Circuit.[27][28][29][25]

III.6 Selective Vulnerability in Frontotemporal Dementia: Clinical Evidence

A particularly powerful line of evidence for the functional importance of VENs comes from neuropathology. In 2006, William Seeley and colleagues at the University of California, San Francisco, published a landmark study demonstrating early, severe, and selective loss of VENs in behavioral-variant frontotemporal dementia (bvFTD). In patients with bvFTD, VEN counts showed a striking 74% reduction compared with control subjects, whereas Alzheimer's disease—which produces profound memory dysfunction without the early social-emotional breakdown characteristic of bvFTD—did not produce significant VEN depletion.[30][31][^32]

This dissociation is clinically meaningful. Behavioral-variant FTD is defined by progressive erosion of social awareness, emotional empathy, self-control, and interpersonal judgment—precisely the domains associated with ACC and FI function. The selective vulnerability of VENs to bvFTD, but not to Alzheimer's disease, implies that VEN loss specifically disrupts the FI–ACC network's contribution to social–emotional conscious awareness, while leaving memory systems—which depend on different circuitry—relatively intact in the early stages. This clinical evidence provides indirect but convergent support for the view that the FI–ACC–VEN axis plays a specialized and non-redundant role in the forms of conscious access most closely tied to

social, emotional, and interoceptive processing—the same domain that Raslan Circuit proposes to explain at the anatomical and biophysical level.[31][33][34][30]

III.7 Contemporary Research: Biochemistry, Connectivity, and Open Questions

The contemporary era of VEN research has extended far beyond morphology and gross anatomy. Immunohistochemical studies have identified a distinctive biochemical profile for VENs, including selective expression of proteins such as ATF3, IL4Rα, and neuromedin B (NMB) that distinguish them from neighboring pyramidal neurons in the same cortical layers. These biochemical signatures provide molecular tools for identifying VENs in experimental settings and raise the possibility that VENs participate in specialized neuromodulatory or immunological signaling pathways not shared by other layer V projection neurons.[^24]

Questions regarding VEN synaptic targets, dendritic morphology in detail, and electrophysiological properties remain incompletely resolved. Whether VENs in FI and ACC share identical or subtly differentiated functional properties is an open question with direct relevance to the Raslan Circuit hypothesis, which assigns complementary transmitter and receiver roles to VENs in these two regions respectively. Progress in high-resolution connectomics, single-cell transcriptomics, and in vivo electrophysiology in animal models with confirmed VEN homologues may offer paths toward resolving these questions in the coming years.[35][29][36][19]

Concluding Note

The three historical threads assembled in this appendix—electromagnetic field theory, antenna engineering, and von Economo neuron research—each followed a trajectory from initial observation or mathematical intuition, through periods of neglect or scepticism, to empirical consolidation and conceptual expansion. Raslan Circuit proposes that the convergence of these threads at the level of VEN morphology, FI–ACC anatomy, and field-based communication principles is not coincidental but reflects a biologically grounded opportunity for a new mechanistic hypothesis. The historical record assembled here is intended to support that convergence argument for readers who wish to evaluate the intellectual foundations of the proposal in depth.

References

1. Electromagnetic wave equation - Wikipedia
2. Milestones:Maxwell's Equations, 1860-1871
3. Maxwell's Field Equations of Electromagnetism - History of Information - An interactive, illustrated timeline of historic moments in humankind's quest for information. With ...
4. Maxwell's Equations of Electromagnetism (1864)
5. [PDF] Lecture 25 Radiation by a Hertzian Dipole - Purdue Engineering
6. File:Hertz spark radio transmitter 1887.png - Wikipedia

7. History | PDF | Antenna (Radio) - This document traces the evolution of antennas from 1886 to 2000 through six examples: Heinrich Hert...
8. Lecture 1 Introduction, Maxwell's Equations
9. [PDF] COPYRIGHTED MATERIAL
10. File:Hertz spark gap transmitter and parabolic antenna.png - Wikipedia
11. Milestones:Reception of Transatlantic Radio Signals, 1901
12. Marconi and the South Wellfleet Wireless - Cape Cod ...
13. File:Marconi Poldhu UK radio transmitter - made first transatlantic transmission 1901.jpg - Wikipedia
14. December 1901 - ITS - Institute for Telecommunication Sciences
15. Profile - Herein, the first English article demonstrating the Yagi-Uda antenna is introduced. The article was ...
16. Yagi antenna design
17. Yagi Uda antenna explained step by step | Know the radiation type of Antenna | Sunday Night Show - It was invented in 1926 by Shintaro Uda of Tohoku Imperial University, Japan, with the help of his ...
18. YAGI UDA antenna .pptx - This document summarizes the Yagi-Uda antenna, which was invented in 1926 by Dr. Shintaro Uda and Dr...
19. Von Economo neurons: Cellular specialization of human limbic ... - Von Economo neurons (VENs) have been mentioned in the medical literature since the second half of th...
20. [PDF] The von Economo neurons in frontoinsular and anterior cingulate ...
21. Von Economo neuron - Wikipedia
22. Spindle Neuron - an overview | ScienceDirect Topics
23. Frontiers | Von Economo Neurons – Primate-Specific or Commonplace in the Mammalian Brain? - The pioneering work by von Economo in 1925 on the cytoarchitectonics of the cerebral cortex revealed...
24. Biochemical specificity of von Economo neurons in hominoids - Von Economo neurons (VENs) are defined by their thin, elongated cell body and long dendrites project...
25. The von Economo neurons in the frontoinsular and anterior cingulate cortex - PubMed - The von Economo neurons (VENs) are large bipolar neurons located in the frontoinsular cortex (FI) an...
26. Total number and volume of Von Economo neurons in the cerebral ... - Our results show that the distribution of VENs in these cetacean species is comparable to that repor...
27. Spindle Neurons in Elephants and Dolphins: Convergent Evolution ... - In cetaceans they can also be found in frontopolar cortex, as in humpback whales (Hof & Van der Guch...
28. Von Economo neurons in the elephant brain - PubMedpubmed.ncbi.nlm.nih.gov › ... - Von Economo neurons (VENs), previously found in humans, all of the great ape species, and four cetac...
29. Evolutionary appearance of von Economo's neurons in ... - Frontiers - VENs are also found in whales (Hof and Van der Gucht, 2007), and in a number of different cetacean...

30. Archive: Study offers window into human behavior, brain disease - UCSF scientists have identified a cell population that is a primary target of the degenerative brain...

31. [PDF] Early frontotemporal dementia targets neurons unique to apes and ...

32. Neurons Targeted By Dementing Illness May Have Evolved For Complex Social Cognition - Von Economo neurons (VENs) are uniquely shaped brain cells that seem to have evolved in a select gro...

33. [PDF] Selective functional, regional, and neuronal vulnerability in ... - von Economo neurons have been shown to represent an early target in the behavioral variant of fronto...

34. Selective Vulnerability of von Economo Neurons in ...

35. Von Economo neurons: Cellular specialization of human limbic cortices? - ## Abstract

Von Economo neurons (VENs) have been mentioned in the medical literature since the seco...

36. Frontiers | Von Economo neurons as a specialized neuron class of the human cerebral cortex - By studying human cortical cytoarchitecture, von Economo noticed large spindle-shaped-neurons within...
