# Supplementary material for: Raslan Circuit: von Economo neurons as biological dipole antenna elements in a proposed FI–ACC communication pathway for multimodal integration and conscious access
Source: Front Syst Neurosci. 2026 Aug 21;20:1779601. doi: 10.3389/fnsys.2026.1779601 (PMC13541754; doi:10.3389/fnsys.2026.1779601)
Supplement: Supplementary file 2 [file Data_sheet_2.pdf]

Supplementary Appendix B – Raslan Circuit, Consciousness Engineering, and the Search for a Concrete Causal Structure

Submitted as Optional Supplementary Material
Raslan Circuit: Von Economo Neurons as Biological Dipole Antenna Elements in a Proposed FI–ACC Communication Pathway for Multimodal Integration and Conscious Access
Frontiers in Systems Neuroscience — Consciousness Engineering Research Topic

Historical and Theoretical Context

Historically, René Descartes sought a single anatomical locus at which organized physical processes could give rise to unified subjective experience, a role he famously—and, in light of contemporary evidence, incorrectly—assigned to the pineal gland.[web:97][web:102] The question itself, however, was not entirely misguided. In modern terms, Descartes was groping toward a structure that could serve as a high-specificity interface between the dynamical organization of the brain and the coherence of conscious experience. Rather than reviving a dualist interpretation, the present work can be viewed as a contemporary, biologically grounded answer to that underlying question.

Raslan Circuit as a Falsifiable Anatomical Candidate

Raslan Circuit proposes the FI–ACC–VEN axis as a biophysically grounded, field-organized, and anatomically specific candidate causal structure through which multimodally integrated, salience-weighted information is assembled and routed toward systems involved in conscious access.[file:1] In this sense, the circuit is not advanced as a complete theory of consciousness, but as a concrete and falsifiable anatomical hypothesis about one critical node in the wider causal chain that supports conscious experience. Its specificity at the level of cortical territory (FI and ACC), cellular substrate (VENs in layer Vb), and putative biophysical mechanism (field-sensitive, antenna-like coupling) distinguishes it from more abstract functional theories that do not identify a particular physical implementation.[web:81]

Position within Consciousness Engineering

The Consciousness Engineering framework, as articulated in the associated Research Topic, treats consciousness as emerging from organized dynamical processes within a physical substrate and frames death as an engineering challenge: how to sustain the causal dynamics that underlie the process-world-line of consciousness across damage, repair, and even substrate transitions.[web:79][web:74][web:81] Progress on this problem requires identifying specific biological structures whose disruption leads to process collapse and whose preservation or biomimetic replication is therefore a necessary condition for continuity. Raslan Circuit offers one such candidate by singling out the FI–ACC–VEN axis as a potentially non-redundant communication infrastructure for a particular class of conscious contents—

those that are multimodally integrated, interoceptively grounded, and behaviorally salient.[file:1]

Gateway to New Empirical and Engineering Avenues

On this reading, Raslan Circuit can be interpreted as a possible "gateway" structure: an open-ended, field-organizing circuit that may generate a characteristic spatiotemporal electromagnetic signature at the interface between deep cortical territories and the surrounding field. Characterizing the geometry, magnitude, and spectral properties of this signature—and testing whether it is preserved, degraded, or reconstructable under targeted perturbations—may open new empirical and engineering avenues for Consciousness Engineering, by providing a concrete, anatomically anchored target for continuity metrics and biomimetic implementation.[web:79][web:103] Whether the field dynamics organized by this axis reflect purely endogenous neural processes or participate in more fundamental organizing principles is left as an open empirical question for future work.
